# Effect of monosodium glutamate on fetal development and progesterone level in pregnant Wistar Albino rats

**DOI:** 10.1007/s11356-023-25661-x

**Published:** 2023-02-14

**Authors:** Hadeer M. Shosha, Hala M. Ebaid, Eman A. Toraih, Heba M. A. Abdelrazek, Ranwa A. Elrayess

**Affiliations:** 1 Zoology Department, Faculty of Sciences, Suez Canal University, Ismailia, 41522 Egypt; 2 Department of Surgery, School of Medicine, Tulane University, New Orleans, LA USA; 3 Genetics Unit, Department of Histology and Cell Biology, Faculty of Medicine, Suez Canal University, Ismailia, 41522 Egypt; 4 Department of Physiology, Faculty of Veterinary Medicine, Suez Canal University, Ismailia, 41522 Egypt

**Keywords:** Ghrelin, MSG, Progesterone, Teratogenicity, Toxicity, Liver, Kidney

## Abstract

Monosodium glutamate (MSG) is a widespread flavor enhancer and stabilizer in manufactured or packaged foods that possess myriad adverse effects. This study aimed to evaluate the effect of MSG on placental progesterone receptors and fetal development. Thirty pregnant Wistar Albino rats were divided into three groups (ten/each). The control group (G1) gavaged distilled water only, low-dose treated group (G2) gavaged 3 g/kg MSG, and high-dose treated group (G3) gavaged 6 g/kg MSG from 1st to 18th days of gestation, and all pregnant rats were sacrificed on the 19th day of gestation. The effect of MSG on fetal weights, crown vertebral length (CVL), placental weight, placental ghrelin expression, and fetal skeleton examination were estimated. MSG induced a significant decrease in fetal weights, CVL lengths, placental weight, and ghrelin expression in both treatment groups compared to the control group. Several parts of the fetal skeleton showed incomplete ossification and delayed chondrification in which high-dose maternally treated fetuses were more affected. Many degenerative changes were detected in both maternal and fetal liver and kidney tissues in MSG-treated groups. Moreover, MSG caused a significant increase in serum ALT, ALP, and creatinine levels in pregnant rats’ blood. Serum progesterone was only elevated in G3 on the 19th day of gestation. This study showed that the administration of MSG during pregnancy adversely influences fetal growth and skeletal development and caused several biochemical and histological changes in the maternal and fetal liver and kidney tissues which assure the toxic and teratogenic effects of MSG.

## Introduction

In the last decade, most people prefer consuming fast foods that contain low fiber but high energy rather than natural or little-treated foods due to changes in lifestyle (Bryant and Dundes 2008, Dongen et al. 2010). Flavors and flavor enhancers are used to bring out the flavor in a wide range of foods without adding a flavor of their own, improving the palatability of the food distinctly (Elhariry et al. 2004). Flavorings are vital in savory food manufacturing and could play an important nutritional role by providing needed appeal (Löliger 2000).

Monosodium glutamate (MSG) is one of the most used flavor enhancers worldwide. It is a non-essential amino acid found in high protein food products such as meat, fish, certain types of cheese and vegetables (Yamaguchi and Ninomiya 2000, Wifall et al. 2006, Shigemura et al. 2009, Kochem and Breslin 2017). MSG has a unique taste (umami) (Kurihara 2015, Stańska and Krzeski 2016). MSG was identified 100 years ago by Kikunae Ikeda which is the fifth basic taste, in addition to sweet, sour, salty, and bitter. In addition to its basic specificity, the umami taste can enhance overall flavor intensity and improve food palatability (Zanfirescu et al. 2019). It is considered a flavoring agent, being used thoroughly in industrialized food. Its production increased from 200.000 tons/year in 1969 to 3 million tons per year being consumed in the global market in 2021, which reflects its crescent use in the food industry (Hermanussen et al. 2006, Nakamura and Itsub 2021). Moreover, MSG is artificially added to food for providing an expansion and extension of taste, whereas it stimulates receptors located in taste buds (Taylor-Burds et al. 2004, Diniz et al. 2005).

The safety and toxicity of MSG have become controversial in the last few years because of reports of adverse reactions in people who have eaten foods that contain MSG. Many studies have confirmed such adverse reactions (Obochi et al. 2009, Meraiyebu et al. 2012). MSG has been reported to cause headaches, vomiting, diarrhea, irritable bowel syndrome, asthma attacks in asthmatic patients, and panic attacks (Hall et al. 1996). Moreover, MSG is shown to have side effects on different body systems; studies reported that diets that contained food additives affected brain neurotransmission, psychological status, behavior, and memory, especially in children (Zeisel 1986, Löwik 1996, Pollitt et al. 1996, Umukoro et al. 2015, Zanfirescu et al. 2019, Akataobi 2020). Moreover, it is believed that MSG can induce obesity; pregnant women who consumed MSG during the gestational period could have a connection with future obesity of the newborn, who suffered from hypothalamic lesions while being in the uterus (Fernández-Tresguerres 2006, Hermanussen et al. 2006).

Administration of any teratogen at the gestation period negatively influenced fetal body weight and differentiation especially when they are administered during the organogenesis period (Carlson 2009). MSG was shown to penetrate placental barrier and distribute almost evenly among embryonic tissues (Gao et al. 1994, Abu Elnaga et al. 2019).

Ghrelin is a hormone that acts as an endogenous ligand for the GH secretagogue receptor (Kojima et al. 1999). This hormone is produced from the stomach, kidney (Mori et al. 2000), hypothalamus (Korbonits et al. 2001), pancreas (Date et al. 2002), and placenta (Gualillo et al. 2001); however, the principal source is the gastrointestinal tract. Placental ghrelin plays a pivotal role in the differentiation and growth of placental cells, modulates growth hormone production and influences fetal development (Nakahara et al. 2006).

Some studies reported the adverse effects of MSG on the female reproductive system (Miskowiak and Partyka 1999, Mondal et al. 2018), but studies of its possible impact on the placental progesterone and fetal development are lacking. Thus, the main aim of the current study was to evaluate the effect of MSG on progesterone level, fetal growth which judged by placental ghrelin expression and skeletal abnormalities in Wistar Albino rats.

## Materials and methods

### Animals

Thirty adult virgin female rats and ten male Wistar Albino rats were used in this study. Their weights ranged from 200 to 220 g. Rats were obtained from the Animal House of the Faculty of Veterinary Medicine, Suez Canal University, Ismailia, Egypt. Animals were kept under standard conditions and allowed free access to food and water. The animals were kept in a natural day-night cycle at room temperature 25 ± 2 °C and kept for 2 weeks for adaptation. The technical standards and international guidelines for research on animals were followed according to the ethical guidelines for animal use in laboratory experiments of the Faculty of Veterinary, Suez Canal University, Egypt (02019020). Only female rats that completed at least two consecutive estrous cycles were used in the present study (Ekambaram et al. 2017). Three females in proestrus were mated with one fertile male per cage overnight. The presence of a vaginal plug or sperm in the vaginal smears the next morning was considered the zero-day of pregnancy (Piesta et al. 2009). Pregnant rats were weighed daily to follow-ups on their pregnancy.

### Monosodium glutamate (MSG)

The MSG was provided in the form of white crystalline powder and fast-soluble in water, supplied by Merck Co., USA (CAS No. 6106-04-3). Monosodium glutamate was dissolved in distilled water, and the dose for each rat was determined according to its respectable body weight.

### Experimental design

Thirty pregnant Wistar Albino rats were divided into three groups ten pregnant rats for each. Control group (G1) gavaged distilled water only, low-dose treated group (G2) gavaged 3 g/kg MSG, and the high-dose treated group (G3) gavaged 6 g/kg MSG according to Eweka and Om’Iniabohs (2007) from the 1st to 18th days of gestation. All pregnant females were observed on daily basis throughout gestation.

### Morphological examination

Maternal body weights were measured day after day, and the percent of maternal body weight gain was calculated according to the following equation:

$$\frac{\mathrm{mean}\;\mathrm{final}\;\mathrm{weight}\;-\;\mathrm{mean}\;\mathrm{initial}\;\mathrm{weight}}{\mathrm{mean}\;\mathrm{initial}\;\mathrm{weight}\;} {\times100\;}$$

All the pregnant rats were sacrificed under tetrahydrofuran inhalation anesthesia on day 19th of gestation, and then uterine horns were removed and freshly photographed. The fetuses were removed from the uterus and cleaned before being weighed and photographed. The uterine shape, number of living and dead fetuses, the fetal crown vertebral length (cm), the fetal weight (g), the placental weight (g), and the presence of external malformations were recorded.

### Skeletal preparation

Skin, viscera, adipose tissue, and eyes were detached from fetuses and kept in 95% ethyl alcohol for 4 days followed by acetone for 1 day to remove the fat. Fetuses were double stained for cartilage and bone (Alizarin red S plus Alcian blue staining), according to Inouye (1976). Then, the stained fetuses were washed with water and placed in 1% aqueous KOH to remove soft tissue and then put ascending series of glycerol and 1% aqueous KOH solution until preserved in 100% glycerin. Skeletal systems of fetuses from different groups were photographed and examined for the presence or absence of ossification centers, and any abnormality of bone was recorded.

### Blood sampling and blood parameters

At the 7th, 14th, and 19th days of gestation, blood samples were collected from the eye using the orbital sinus technique (Sanford 1954). The blood was collected and allowed to clot, and then the serum was separated by centrifugation at 3000 rpm for 20 min, and the clear non-hemolyzed serum was collected, divided into several aliquots, and stored at – 80 °C until assayed. Serum alkaline phosphatase (ALP) was measured using a kit provided by Abcam (Cat. No ab83369, UK), while alanine transaminase (ALT) was determined using a kit provided by DIACHEM Ltd. (Cat. No. 48261, Hungary). Serum creatinine was determined by a kit provided by DIACHEM Ltd. (Cat. No. 46161, Hungary), while serum progesterone was determined by progesterone ELISA kit provided by TECAN (REF52231, Germany).

### Histopathological preparation

At the 19th day of gestation, samples of the liver, kidney, and placenta of pregnant mothers beside the fetal liver and kidney were collected and fixed in 10% neutral buffer formalin for the histological techniques. Then, they were dehydrated in ascending series of alcohol after washing, cleared in xylene, and embedded in paraffin wax. The paraffin blocks were cut 5 μm in thickness and mounted on glass slides stained with hematoxylin and eosin (Drury and Wallington 1980).

### Placental ghrelin expression

Ghrelin gene expression was assessed in rat tissues. Samples of feto-maternal junctions and placentae of day 7, day 14, and day 19 of pregnant rats from the control and MSG-treated groups were collected in RNA stabilizing reagent. Total RNA was extracted using an RNeasy minikit (Qiagen Co, cat no 74104, USA) following the instructions of the manufacturer. Samples were subjected to RNase-free DNase I treatment for 2 h at 37 °C. Next, RNA purity and concentration were assessed using a NanoDrop ND-1000 spectrophotometer (NanoDrop Tech, USA). For reverse transcription reaction, RNA samples (10 ng) and a high-capacity cDNA Reverse Transcription Kit (Applied Biosystems, USA) were used. Reactions were carried out in a Mastercycler Gradient Thermocycler (Eppendorf, Germany). Real-time PCR was employed following the Minimum Information for Publication of Quantitative Real-Time PCR Experiments (MIQE) Guidelines. The ghrelin gene was analyzed in the fetomaternal and placental tissues and then normalized to beta-actin gene with accession number “V01217” according to Langnaese et al. (2008). Primer pairs were obtained from ORIGENE Company (USA). Table 1 represents the reaction containing the cDNA template, qPCR Green Master (Jena Bioscience, Germany), forward and reverse primers, and PCR-grade water was run in StepOne Real-Time PCR system (Applied Biosystems, USA), as previously described.

**Table 1:** Primer pairs of genes for real-time PCR analysis

Gene	Forward	Reverse
Gherlin	CCAGAGGACAGAGGACAAGC	AGTTGCAGAGGAGGCAGAAGCT
ACTB	AAGTCCCTCACCCTCCCAAAAG	AAGCAATGCTGTCACCTTCCC

The fold change of mRNA expressions in each tissue sample was calculated relative to the control tissues via Livak and Schmittgen (2001) method based on the quantitative cycle (Cq) values with the following equation: relative quantity = 2^−ΔΔCq^.

### Statistical analysis

Data were analyzed using one-way analysis of variance (ANOVA) followed by Duncan’s (IMB-SPSS version 28.0 for Mac OS) for comparing the means value obtained in the different groups (Knapp 2017). Data were represented as mean ± SE, and significance was defined as *P* < 0.05. Being non-parametric, a logistic transformation of gene expression was used. A two-way ANOVA test with the Geisser-Greenhouse correction was employed to estimate how the gene expression changes according to the levels of MSG dose and the time point of sampling simultaneously. The corrected method of Benjamini and Yekutieli was computed for each comparison. A *p* < 0.05 was considered statistically significant.

## Results

### Morphological studies

**Fig. 1:**
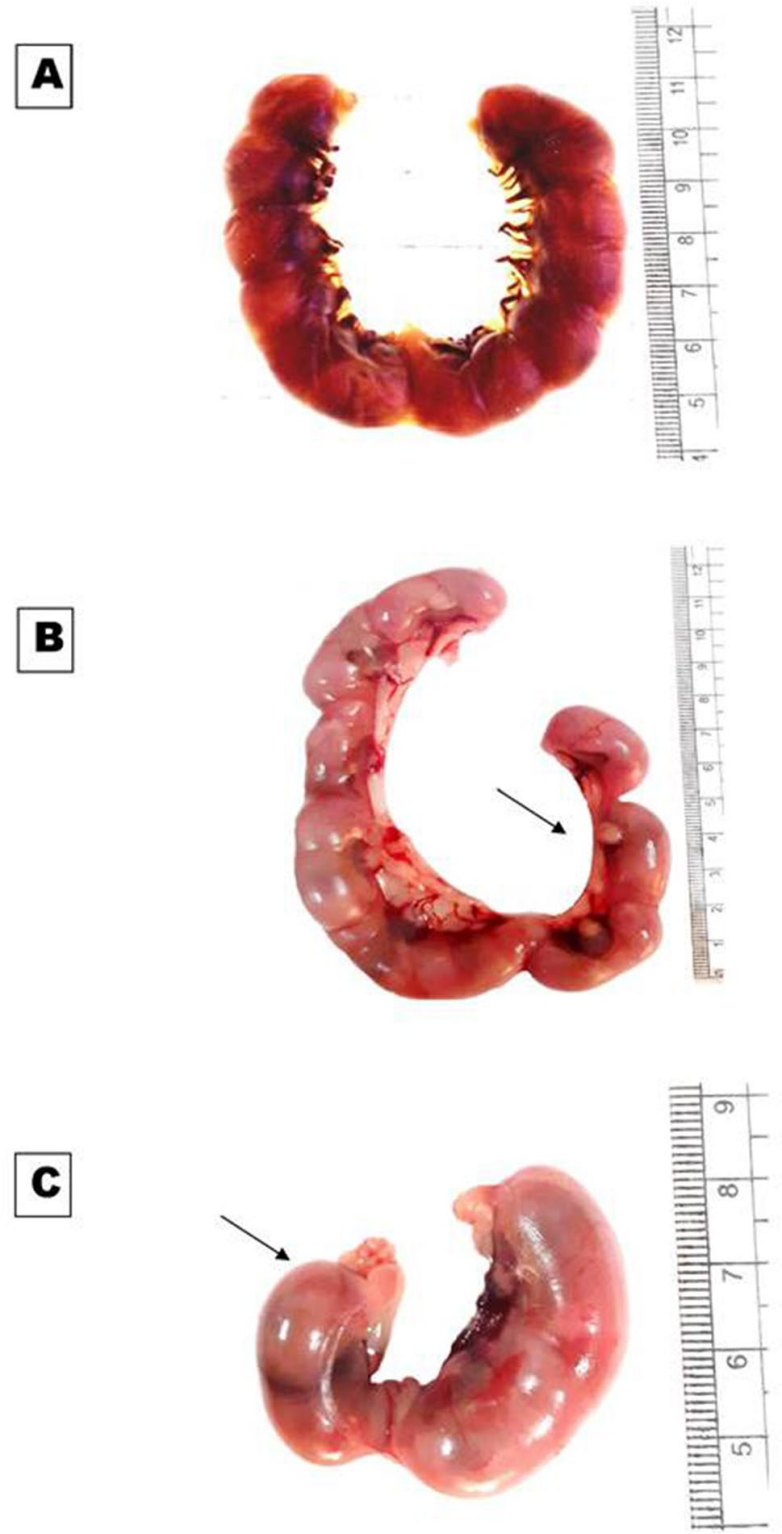
Photographs of the uteri of pregnant rats at the 19th day of gestation showing: **A** uterus of control rat with equal distribution of implantation sites on the uterine horns. **B, C** Uterus of treated rats with low and high doses of MSG respectively, showing the unequal distribution of implantation sites on the uterine horns (arrow)

The result indicated that there was a steady increase in the body weight of all pregnant rats in different groups during the gestation period. The average percentage of increase in maternal weight at the 19th day of gestation was significantly higher in treated groups than in control ones (Table 2). All females in the control, low, and high-dose groups have completed their pregnancy without any abortion cases; no deaths were recorded in the control or treated groups. However, MSG altered the distribution of implantation sites in the uterine horns in all treated groups (Fig. 1). Moreover, the placental weights of maternally treated fetuses were significantly decreased compared to the control. The total number of fetuses per dam showed a significant decrease as compared with controls; it was minimal in the high-dose group (Table 2).

**Table 2:** Effect of MSG on the average increase in maternal body weight gain (g), fetus length and weight, placental weight, and fetus morphology and skeleton at the 19th days of gestation

Parameters	Control	Treated	
		Low dose	High dose
Initial body weight	204 ± 2.0 ^a^	204.00 ± 1.9^a^	209.22 ± 6.7^a^
Final body weight	227 ± 1.5^b^	247.6 ± 8.0^a^	253 ± 7.5^a^
Maternal body weight gain	23 ± 1.6 ^b^	43.6 ± 7.2^a^	43.78 ± 6.7^a^
% of body weight gain	12 ± 1.0 ^b^	21.45 ± 3.4^a^	21.5 ± 3.6^a^
Total number of mother	10	10	10
Total number of fetuses	87 ^a^	55^b^	34^c^
Mean number/dam	8.7 ± 0.27^a^	5.5 ± 0.26^b^	3.4 ± 0.23^c^
Fetus length	3.70 ± 0.048^a^	3.44 ± 0.056^b^	3.31 ± 0.067^b^
Fetus weight	4.53 ± 0.12^a^	3.76 ± 0.079^b^	3.62 ± 0.12^b^
Placental weight	0.65 ± 0.022^a^	0.51 ± 0.017^b^	0.47 ± 0.008^b^
kinky tail	0^a^	4 (7.27%) ^b^	9 (26.47%) ^c^
Clubbed forelimbs	0^a^	3 (5.45%)^b^	11 (32.35%)^c^
Soft skin	0^a^	8(14.5%)^b^	20 (58.8%)^c^
Skeletal defects	0^a^	9(16.36%)^b^	34 (100%)^c^

Data represent the mean value ± S.E. from10 rat/group. Mean values with different superscript letters within the same row are significantly different at *P* ≤ 0.05 using ANOVA followed by the Duncan’s multiple comparison test

**Fig. 2:**
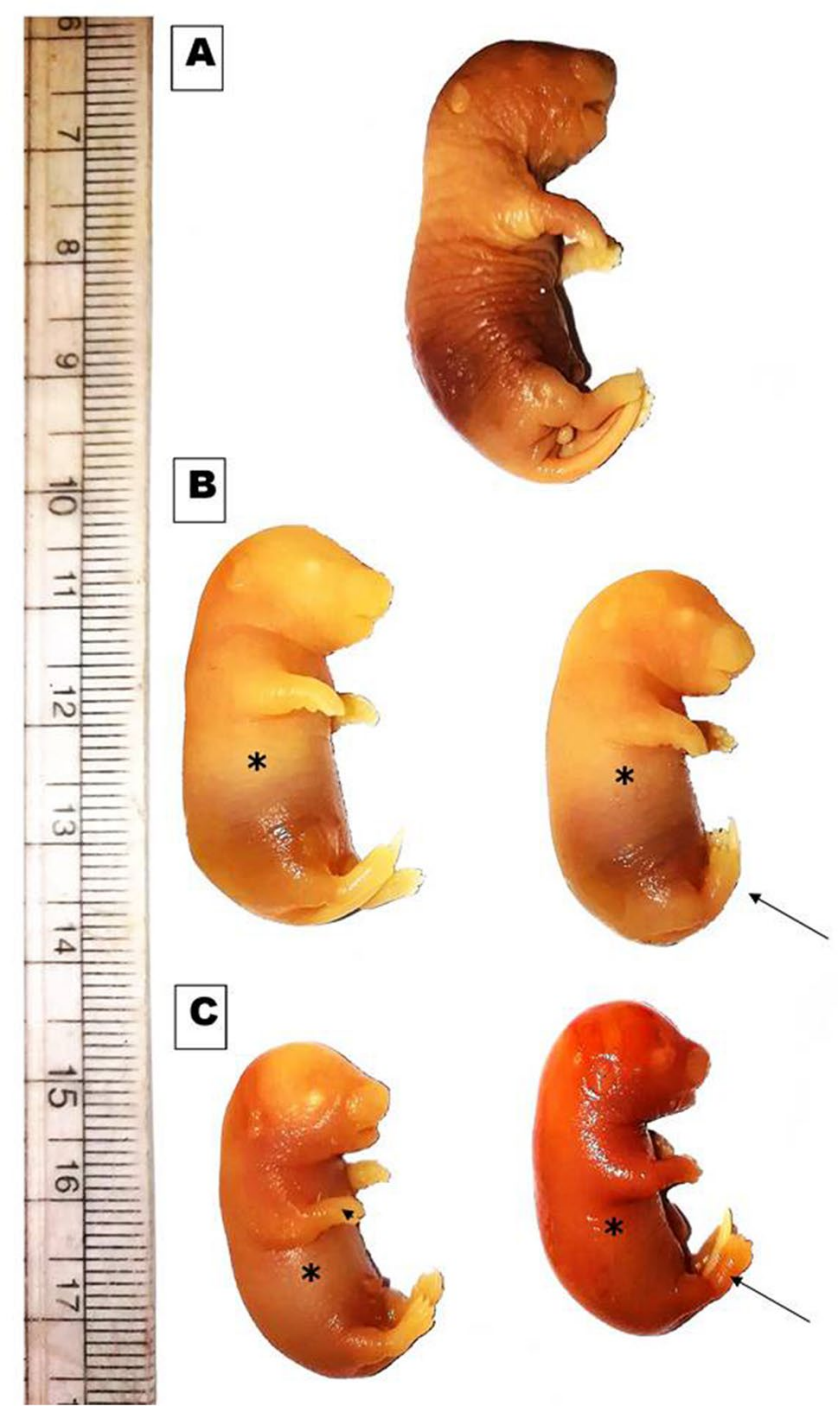
Photomacrograph of the gross morphology of the rat fetuses maternally treated with low and high doses of MSG compared with control, showing: **A** control showing fetus with normal external morphology. **B** Low dose of MSG treatment, showing fetuses with soft membranous skin (*) and kinky tail (black arrow). **C** High dose of MSG treatment, showing fetuses with soft membranous skin (*) and kinky tail (black arrow) beside clubbed fore limb (head arrow)

The morphological examination of fetuses maternally treated with a low or high-dose of MSG showed that some fetuses exhibited soft, membranous, and thin skin and kinky tail as well and others showed clubbed forelimb (Table 2, Fig. 2). Moreover, the weight and length of maternally treated fetuses were significantly decreased compared to their control. The minimal weight and length were observed among fetuses maternally treated with high-dose of MSG (Table 2).

### Skeletal malformation of 19th-day rat fetuses

**Fig. 3:**
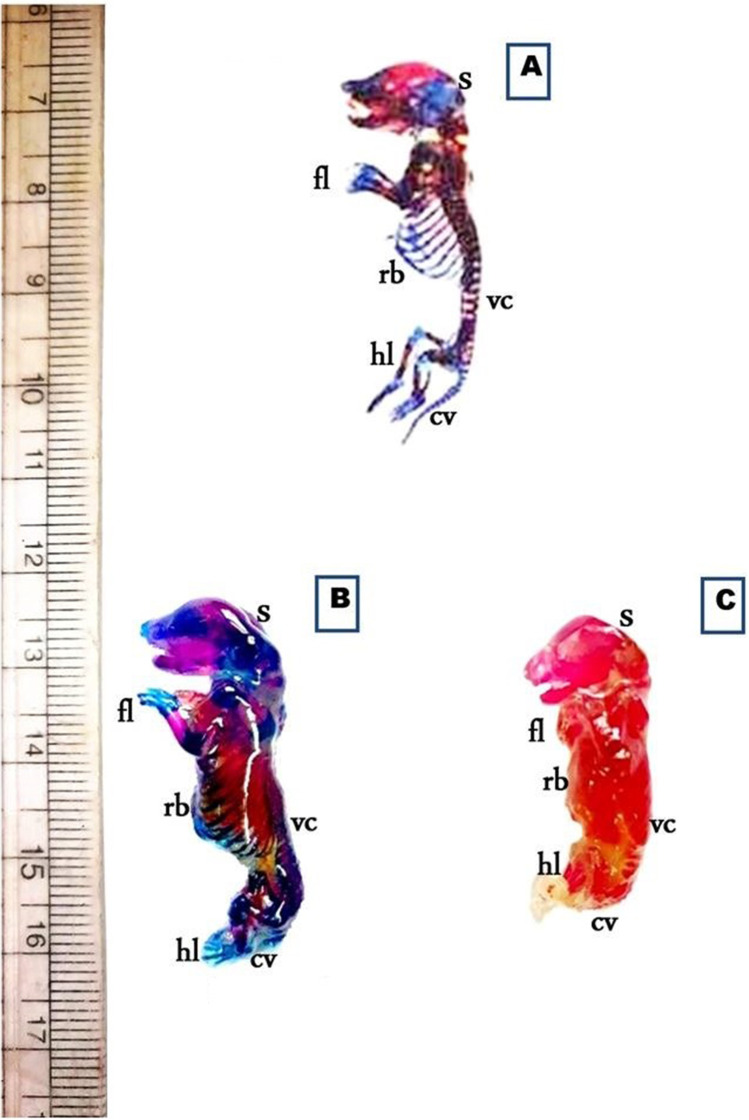
Photomacrograph of the 19th day of gestation full fetal rat skeleton double stained with Alizarin red S and Alcian Blue stains, showing differentiation of bone and cartilage (stain bone red and cartilage blue). **A** Control. **B** Rat fetuses maternally treated with a low-dose of MSG. **C** Rat fetuses maternally treated with a high dose of MSG. Skull (s), forelimb (fl ), ribs (rb), vertebral column (vc), hindlimb (hl), and caudal vertebra (cv)

**Fig. 4:**
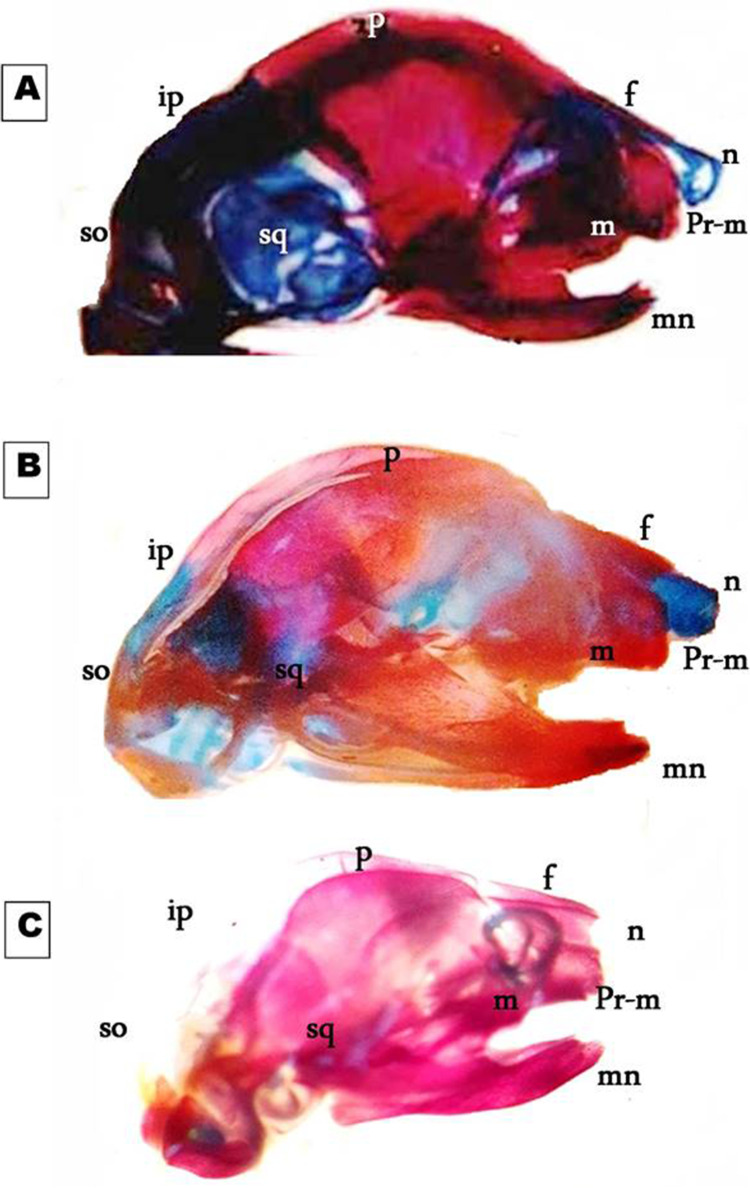
Photomicrographs of a lateral view of the skull of the 19th day of gestation rat fetuses. **A** Control skull showing normally developed nasal (n), frontal (f), parietal (p), interparietal (ip), squamosal (sq), supraoccipital (so), premaxilla (pr-m), maxilla (m), and mandible (mn). **B** Skull of the fetus maternally treated with low dose MSG showing that cartilaginous and bony parts were faintly stained. **C** Skull of the fetus maternally treated with high-dose of MSG showing the absence of cartilage formation in all parts of the skull and delay in ossification of parietal (p) and interparietal (ip) bones. Note the reduction in skull size (8×)

**Fig. 5:**
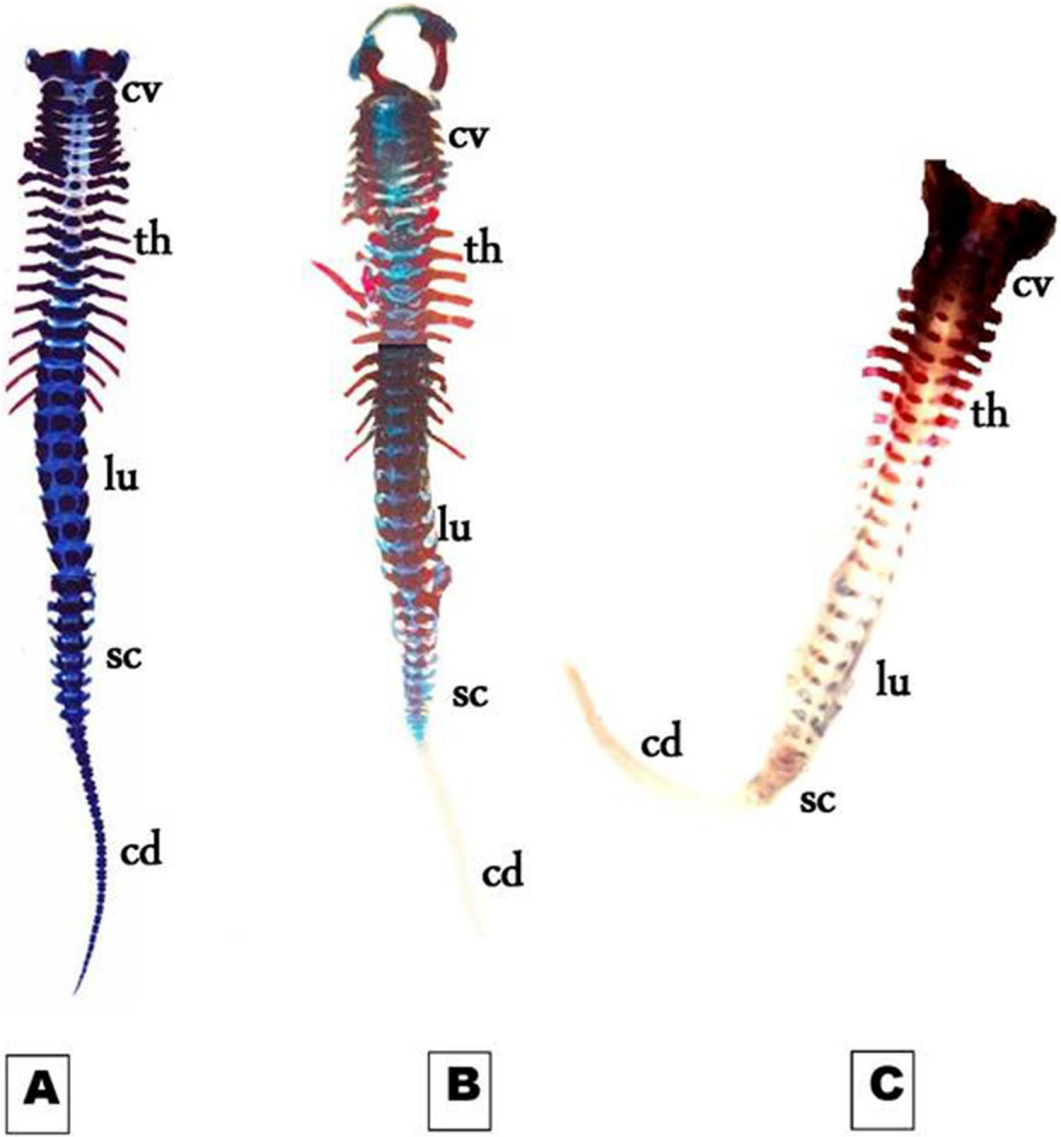
Photomicrographs of a ventral view of the vertebral column of the 19th day of gestation rat fetuses. **A** Control vertebral column showing well-ossified centra and parts of the neural arches of the cervical (cv), thoracic (th), lumbar (lu), sacral (sc), and the first third of caudal vertebrae (cd). **B** Vertebral column of fetus maternally treated with a low dose of MSG showing the absence of chondrification and ossification of the most caudal vertebra (cd). **C** Vertebral column of the fetus maternally treated with a high dose of MSG showing the absence of chondrification in all vertebrae and delay in ossification of the centra of the cervical vertebrae. Note that there is no ossification and chondrification of most caudal vertebrae (6.3X)

**Fig. 6:**
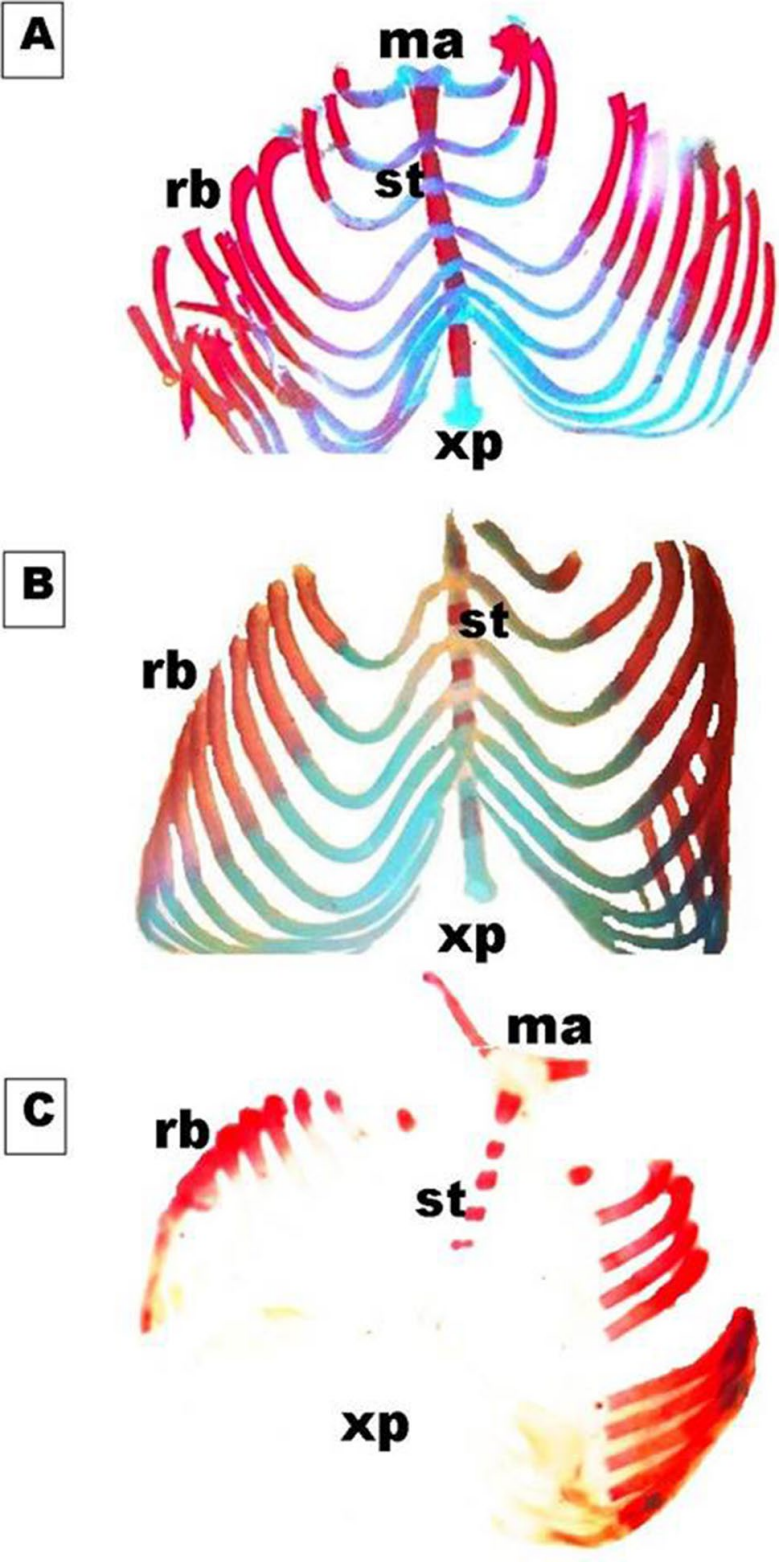
Photomicrographs of a ventral view of the sternum of the 19th day of gestation rat fetuses. **A** Control sternum showing six fully ossified sternebrae (st), the cartilaginous sternal portion of ribs (rb), manubrium (ma), and xiphoid process (xp). **B** Sternum of fetus maternally treated with a low dose of MSG showing faintly stained cartilaginous parts of sternebrae (st) and a sternal portion of ribs (rb). **C** Sternum of fetus maternally treated with a high dose of MSG showing complete delay in chondrification in both sternum and ribs besides the absence of xiphoid cartilage (xp) (7X)

**Fig. 7:**
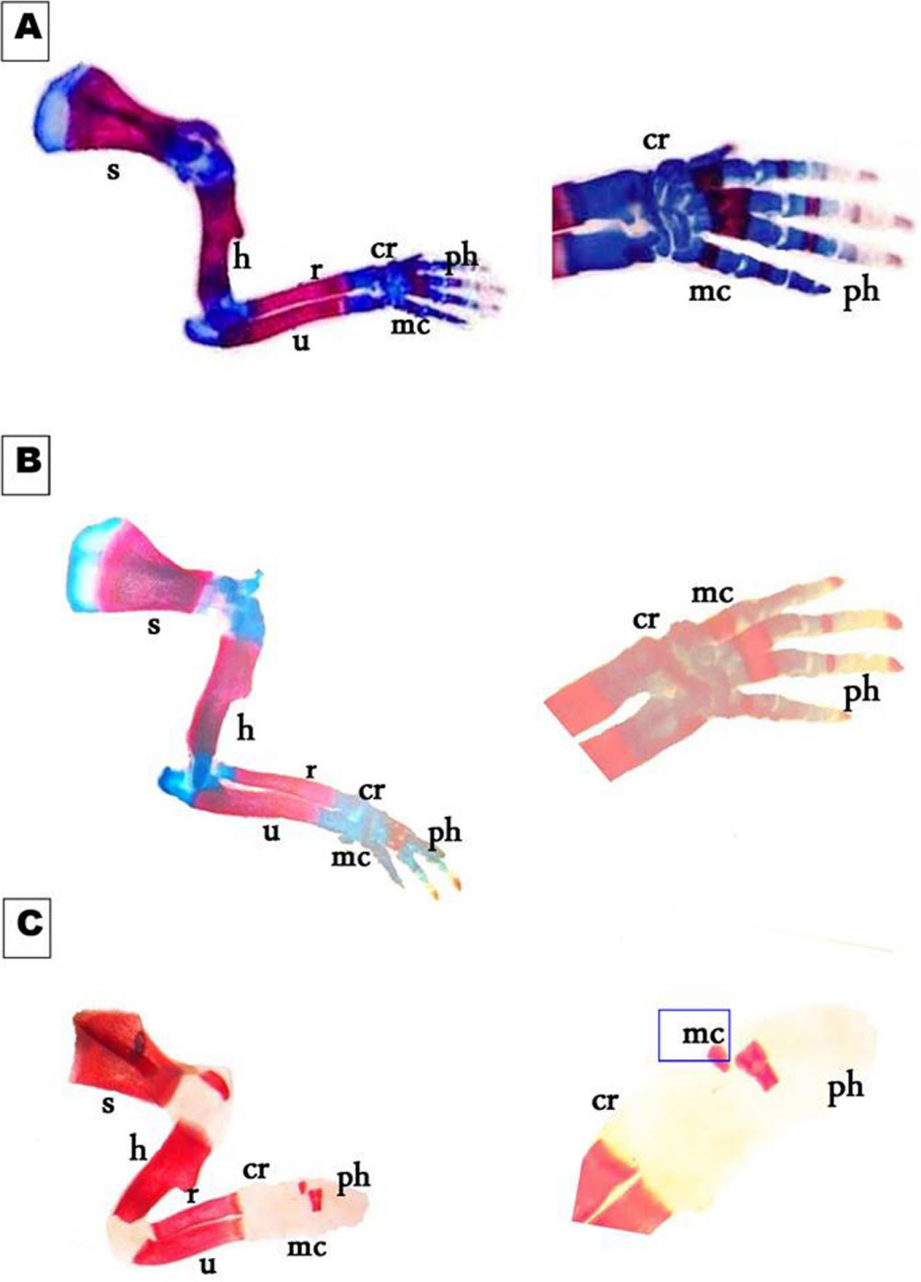
Photomicrographs of a lateral view of the scapula and fore limb of the 19th day of gestation rat fetuses. **A** Control scapula (s) and fore limb showing, fully developed humerus (h), ulna (u) radius (r), carpals (cr), metacarpals (mc), and phalanges (ph). **B** The scapula and fore limb of the fetus were maternally treated with a low dose of MSG showing a normal appearance but faintly stained both bony (red) and cartilaginous (blue) parts. **C** The scapula and fore limb of the fetus maternally treated with a high dose of MSG showing the absence of chondrification in the pectoral girdle and limb besides a reduction in metacarpals size and absence of ossified phalanges (10X, 16 X)

**Fig. 8:**
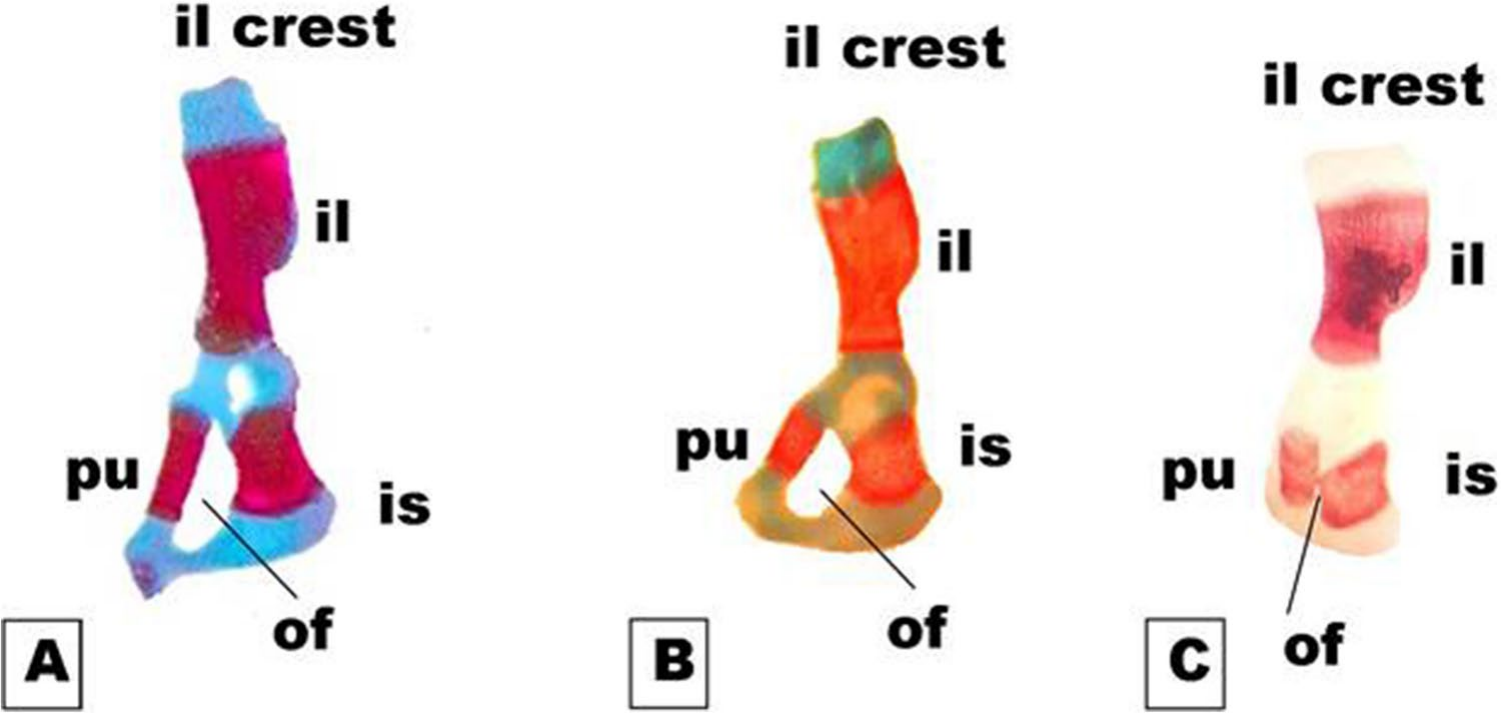
Photomicrographs of a dorsal view of the half of the pelvic girdle on the 19th day of gestation rat fetuses. **A** Control half pelvic girdle showing, well-developed ilium (i), ischium (is), and pubis (pu). **B** The half pelvic girdle of the fetus maternally treated with a low dose of MSG showed a normal appearance but with faint staining of both bone and cartilage. **C** The half pelvic girdle of the fetus maternally treated with a high dose of MSG showing a complete absence of chondrification and altered size of the ileum (il), ischium (is), and pubis (pu) (appear thicker and shorter than normal) and delayed obturator foramen (of) (10X)

**Fig. 9:**
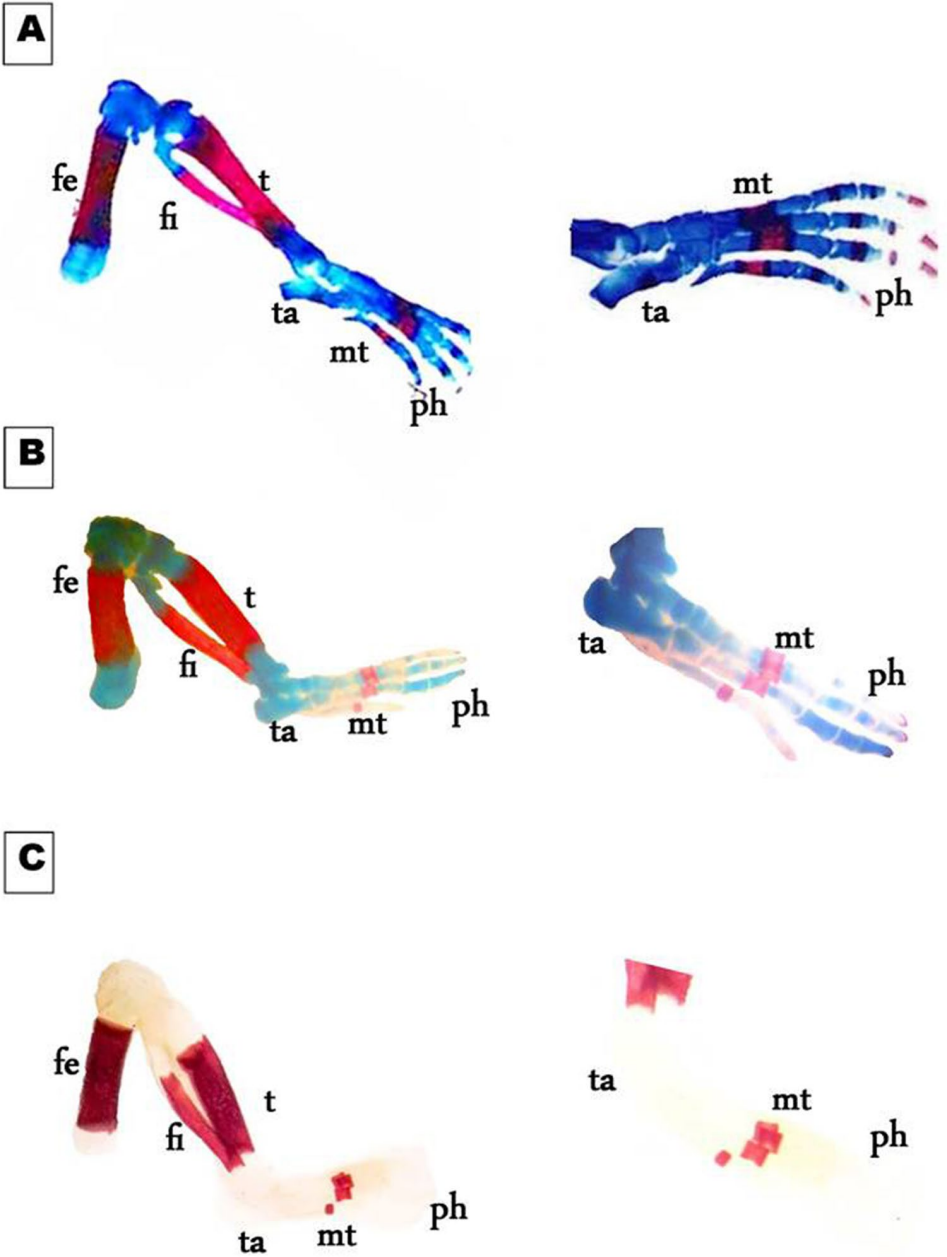
Photomicrographs of a lateral view of the hind limb of the 19th day of gestation rat fetuses. **A** Control hind limb showing, fully developed cartilage and bone of femur (fe), tibia (t), tarsals (tr), metatarsals (mt), and phalanges (ph). **B** Hind limb of the fetus maternally treated with a low dose of MSG shows a delay in chondrification of tarsals (ta) and phalanges (ph). **C** Hind limb of the fetus maternally treated with a high dose of MSG shows the complete absence of chondrification in the entire limb besides the absence of ossified phalanges (ph) (10X, 16 X)

On day 19th of gestation, the skeletal system of a control rat showed that ossification and chondrification processes were completed. However, fetuses maternally treated with MSG showed a marked retardation ossification and delayed chondrification in some parts of the skull, sternebrae, vertebrae, pelvic girdle, pectoral girdle, fore, and hind limbs (Table 2, Fig. 3). Examination of the skull of high-dose maternally treated fetuses’ revealed absence of cartilage formation in all parts of skull (Fig. 4), while the vertebral column exhibited absence of chondrification in all vertebrae and delay in ossification of the center of the cervical vertebrae and absence of most or all caudal vertebrae (Fig. 5). The sternum exerted delay in chondrification of the sternal portion of ribs and absence of xiphoid cartilage (Fig. 6), while complete delay in chondrification of the pectoral girdle and forelimb and absence of ossified phalanges were also recorded (Fig. 7). The pelvic girdle showed absence of chondrification and a great reduction in the size of the obturator foramen (Fig. 8), and the hind showed a complete absence of chondrification in the entire limb (Fig. 9).

### Biochemical parameters

The result showed that serum ALT and creatinine were significantly (*p* < 0.05) elevated in pregnant rats treated with low and high doses of MSG compared to control after the 14th and 19th days of gestation (Table 3). However, serum ALP was significantly (*p* < 0.001) increased in pregnant rats treated with low-dose MSG on the 19th day of gestation only. However, ALP was significantly (*p < 0.001*) increased in the 14th and 19th day of gestation in the high-dose MSG-treated pregnant rats (Table 3).

**Table 3:** Changes in alanine transaminase activity (U/I), alkaline phosphatase (U/I), creatinine level (U/I), and serum progesterone level (ng/Ml) of pregnant Wister albino rats after treating with low and high doses of MSG at different days of gestation

	Time (days)	Control	Treated	
			Low dose	High dose
Alanine transaminase activity (U/I)	14th day of gestation	22.33 ± 1.84^c^	32.3 ± 3.63^bc^	42.36 ± 9.87^b^
	19th day of gestation	23.06 ± 2.19^c^	50.18 ± 10.93^ab^	60.97 ± 8.45^a^
Alkaline phosphatase (U/I)	14th day of gestation	195.01 ± 15.96^c^	200.0 ± 12.41^c^	400.66 ± 42.87^a^
	19th day of gestation	148.93 ± 7.04^c^	203.78 ± 6.78^c^	306.0 ± 20.94^b^
Creatinine level (U/I)	14th day of gestation	0.8 ± 0.23^d^	1.83 ± 0.10b^c^	1.57 ± 0.12^ab^
	19th day of gestation	1.09 ± 0.25^cd^	1.91 ± 0.17^a^	1.78 ± 0.16^ab^
Serum progesterone level (ng/Ml)	7th day of gestation	54.0 ± 2.30^a^	40.0 ± 1.70^b^	31.0 ± 3.50^c^
	14th day of gestation	62.5 ± 3.00^a^	38.2 ± 0.80^b^	46.5 ± 1.50^c^
	19th day of gestation	31.0 ± 3.50^a^	34.5 ± 1.50^a^	70.25 ± 2.00^c^

Data represent the mean value ± S.E. from10 rat/group. Mean values with different superscript letters within the same row are significantly different at *P* ≤ 0.05 using ANOVA followed by the Duncan’s multiple comparison test

Moreover, there was a significant (*p* < 0.05) decrease in serum progesterone levels in pregnant rats treated with a low and high dose of MSG than control after the 7th and 14th days of gestation. However, progesterone level significantly (*p* < 0.05) increased only in the serum of pregnant mothers treated with high-dose MSG at the 19th day of gestation (Table 3).

### Histological studies

**Fig. 10:**
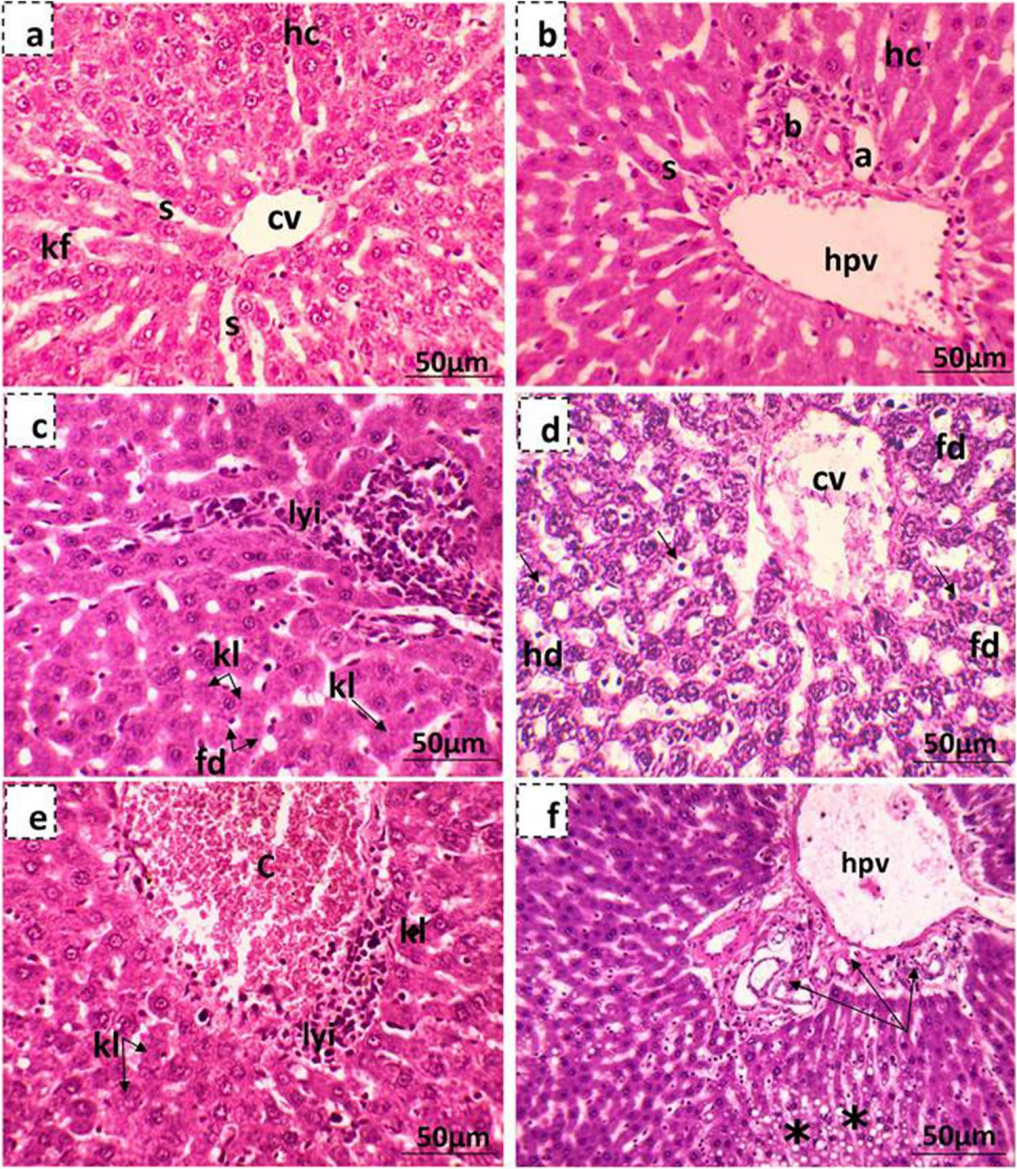
Photomicrographs showing liver tissue of the 19th day of gestation mother rats stained with hematoxylin and eosin. **a, b** Liver tissue of the control mother showing the central and portal areas which contain a branch of the central vein (c), cords of hepatocytes (H), hepatic sinusoids (s), the hepatic portal vein (hpv), branch of the hepatic artery (a), and bile ducts (b). **c, d** Maternal liver tissue of low-dose MSG-treated group where (**c**) shows focal lymphocytic infiltration (lyi), fatty degeneration (fd), Karyorrhexis (kh), and karyolysis (kl) of hepatocytes and (**d**) shows diluted sinusoids and most of the hepatocytes showing hydropic degeneration (hd), fatty degeneration (fd), karyorrhexis (kh), and karyolysis (kl). Some hepatocytes appear with pyknotic nuclei (arrow). **e, f** Maternal liver tissue of high-dose MSG-treated group where (**e**) shows congested central vein (c) with lymphatic infiltration (lyi) and a lot of karyolysis (kl) hepatocyte and (**f**) shows hyperplasia of bile ducts (arrow) with infiltration of inflammatory cells and destruction of the area surrounding the congested portal vein (hpv) and focal fatty degeneration of hepatocytes (star)

**Fig. 11:**
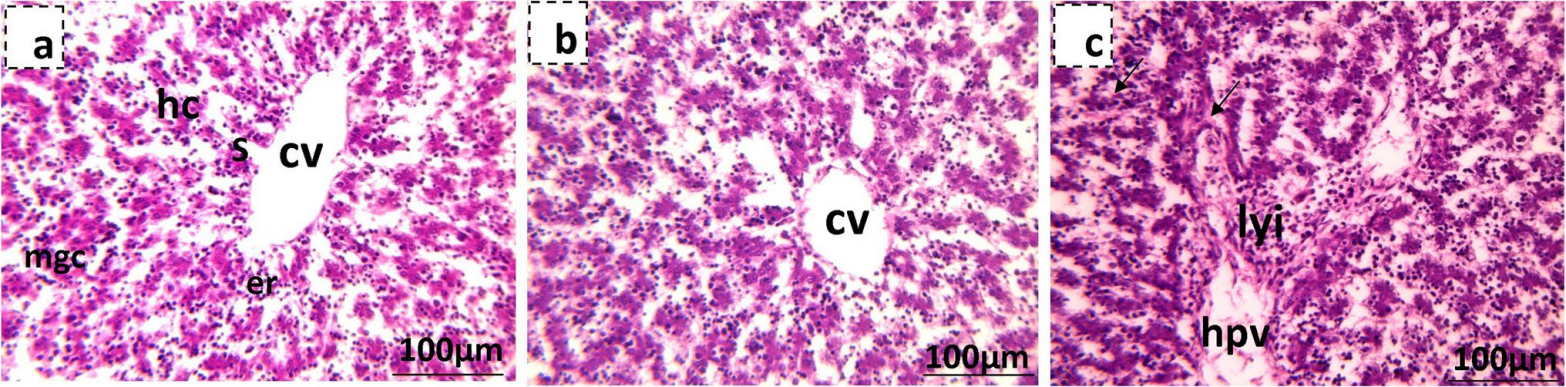
Photomicrographs showing fetal liver tissue of the 19th day of gestation rat fetuses stained with hematoxylin and eosin. **a** Control showing normal histology of fetal liver tissue showing the central vein (cv) surrounded by the hepatocytes (hc), blood sinusoids (s), and megakaryocytes (mgc). **b** Liver tissue of low-dose MSG maternally treated fetus with normal hepatic structure. **c** Liver tissue of high-dose MSG maternally treated fetus showing congested hepatic portal vein (hpv) and sinusoids (arrow) besides lymphocytic infiltration (lyi)

Liver of control pregnant rats showed the normal structure of hepatic tissue with normal central and portal areas. The hepatic tissue consists of plates of polyhedral hepatocytes radially arranged around a central vein and separated by blood sinusoids. The portal areas are arranged at the peripheral angles of each lobule and contain branches from the hepatic portal vein, hepatic artery, and the bile duct (Fig. 10a,b). Microscopic examination of the liver sections of mothers treated with MSG showed different histopathological changes, which were more severe in rats given the high dose of MSG. Histological alternations included both degeneration and necrosis of some hepatocytes. Degeneration was of the hydropic and fatty types. Other hepatocytes showed different shapes of hepatic necrosis, including karyorrhexis, karyolysis, and pyknosis. Besides, dilated sinusoid and lymphocytic infiltration of inflammatory cells surrounding the portal area were also reported (Fig. 10c,d,e). Hyperplasia of bile ducts with infiltration of inflammatory cells and destruction of the area surrounding the portal veins were only noted in the liver section of high-dose MSG-treated mothers (Fig. 10f). The hepatic tissues of the 19th day of gestation control rat fetuses are composed of irregular hepatic plates formed of hepatocytes. Between the cords, sinusoids which are irregular, dilated, and lined with a discontinuous layer of endothelial and Kupffer cells are found. Numerous young, nucleated erythroblasts are found inside the sinusoids and in between the hepatic cords. Few megakaryocytes are encountered in blood sinusoids of control fetus rat liver. The liver examination of low-dosed maternally treated fetuses showed normal hepatocytes and liver structure (Fig. 11a), while the liver of highly dosed maternally treated fetuses showed hydropic degeneration, and the central and portal veins as well as blood sinusoids were congested; besides, lymphocytic infiltration was also observed (Fig. 11b,c).

**Fig. 12:**
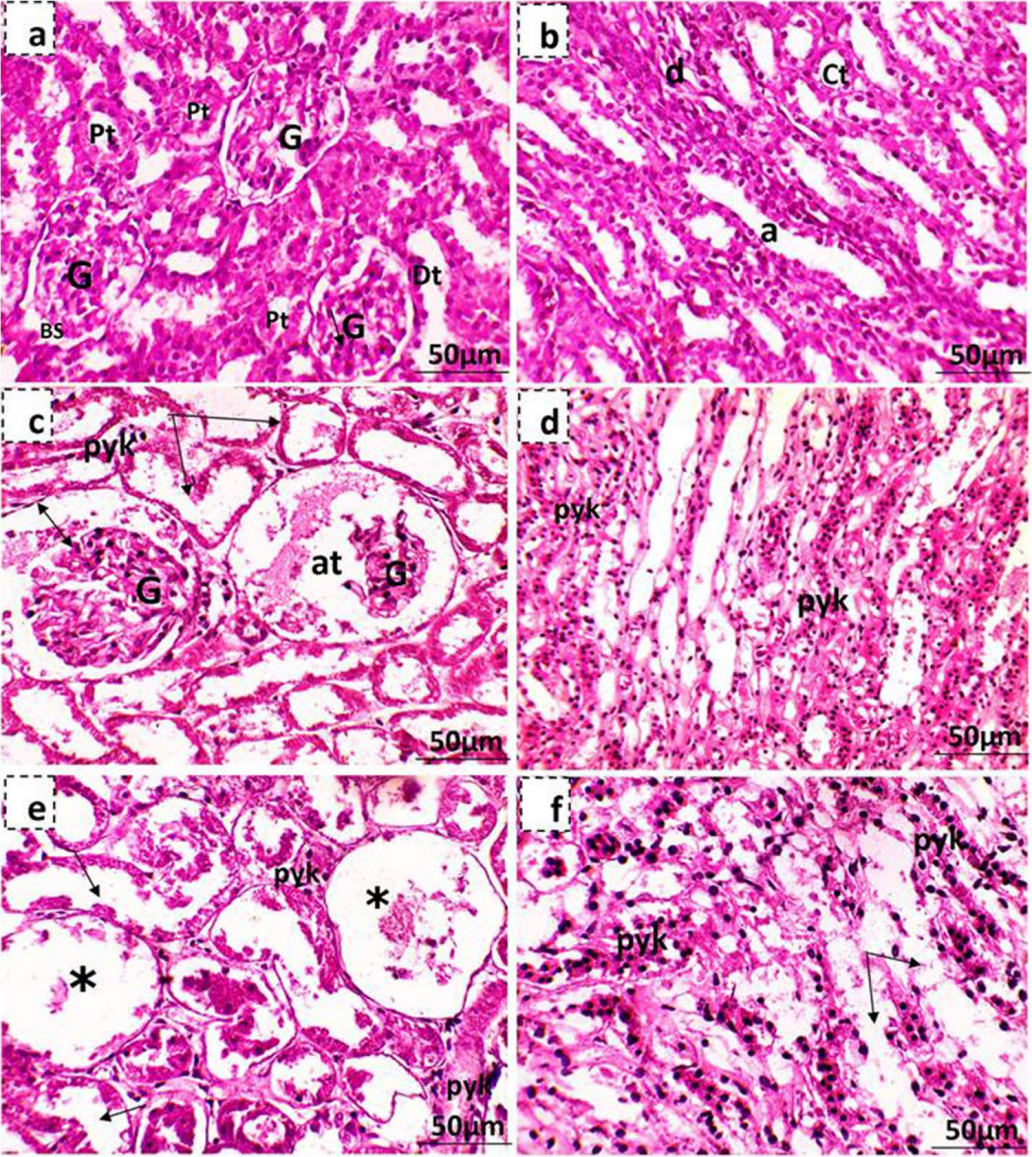
Photomicrographs showing kidney tissue of the 19th day of gestation mother rats stained with hematoxylin and eosin. **a, b** Kidney tissue of the control mother showing normal histological structures of renal corpuscles with its glomerulus (G), Bowman’s space (BS), and portions of the proximal (pt) and distal (dt) convoluted tubules, while the medullary region contains the descending (d) and ascending (a) limbs of Henle’s loop and the collecting tubules (ct). **c, d** Maternal kidney tissue of low-dose MSG-treated group where (**c**) shows atrophied renal corpuscle (at), dilatation of the urinary space (↔) and degeneration of cells lining renal convoluted tubules (arrow) and other cells were pyknotic (pyk) and (**d**) shows pyknosis of some epithelial cells lining the descending and ascending limbs of Henle’s loop and the collecting tubules (pyk). **e, f** Maternal kidney tissue of high-dose MSG treated group where (**e**) shows the cortical region containing renal corpuscles with completely degenerated glomeruli (*) and pyknosis (pyk) beside degenerated convoluted tubules (arrow) and (**f**) shows the medullary region pyknosis and degenerated descending and ascending limbs of Henle’s loop and the collecting tubule (arrow)

**Fig. 13:**
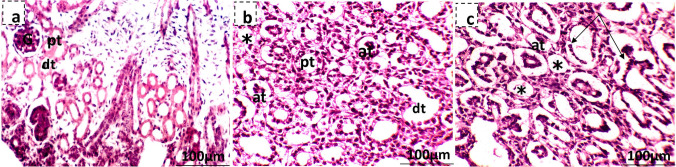
Photomicrographs showing fetal kidney tissue of the 19th day of gestation rat fetuses stained with hematoxylin and eosin. **a** Control showing normal histology of fetal kidney tissue showing kidney capsule (cp), Bowman’s capsule (bc), and glomerulus (gl) portions of the proximal (pt) and distal (dt) convoluted tubules. **b** Kidney tissue of low-dose MSG maternally treated fetus showing kidney tissue lost its normal configuration, many atrophied renal corpuscles (at), and others degenerated (*). **c** Kidney tissue of high-dose MSG maternally treated fetus showing many atrophied renal corpuscles (at) and others with completely degenerated glomeruli (*) beside shedding out of epithelial lining the renal convoluted tubules (arrow)

Kidneys of control pregnant rats showed normal structure with regular cortex and medulla areas (Fig. 12a,b). The histopathological examination of low-dose MSG-treated mother’s kidney showed some atrophied renal corpuscle, and the epithelia of the visceral and parietal layers of some Bowman’s capsules have degenerated with dilatation of the urinary space. Besides, some cells lining the proximal and distal convoluted tubules were pyknotic, and others were degenerated (Fig. 12c,d). However, the high-dose MSG-treated mothers showed cortical regions with many atrophied and degenerated renal corpuscles, besides pyknosis and degenerated convoluted tubules, while the medullary region showed pyknotic and degenerated descending and ascending limbs of Henle’s loop and the collecting tubule (Fig. 12e,f). The kidney of the 19th-day control fetus rats was differentiated into cortex and medulla closely covered with connective tissue. The cortex contains Malpighian corpuscles and proximal and distal tubules. In contrast, the medulla contains undifferentiated mesenchymal cells and is occupied by the ascending and descending loops of Henle (Fig. 13a). The kidney examination of MSG maternally treated fetuses showed that kidney tissue lost its typical configuration; some glomeruli were atrophied, and others with degenerated glomeruli (Fig. 13b), while some renal tubules appear deteriorated (shedding out of epithelial lining the convoluted renal tubules) (Fig. 13c).

### Ghrelin expression in placenta

**Fig. 14:**
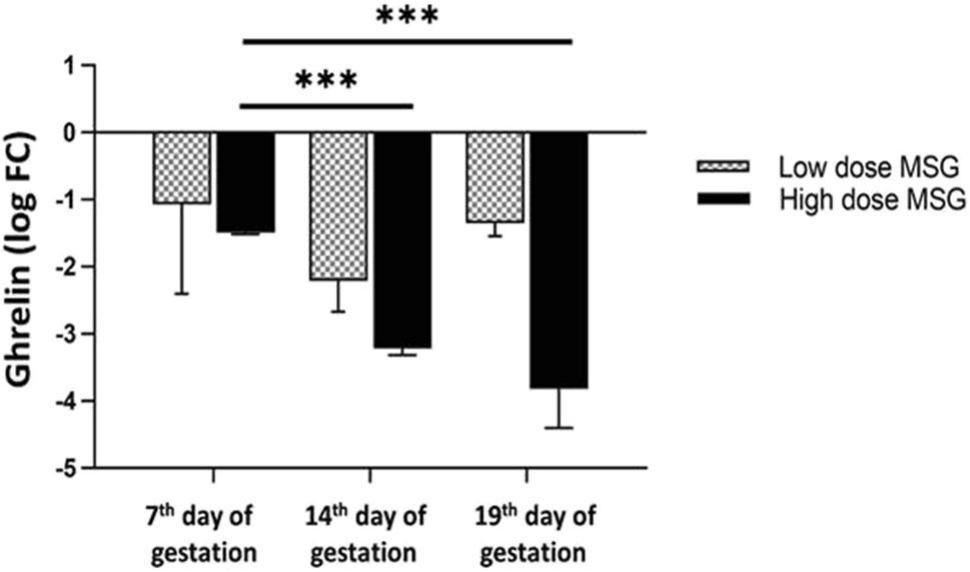
Relative expression of the ghrelin gene in the placenta. Logarithmic transformation of fold change was used. A two-way ANOVA test was applied. ***p < 0.001

Compared to control samples, MSG treatment was associated with significant suppression of ghrelin gene expression in the placenta at days 7, 14, and 19 groups compared to their corresponding control group (*p* < 0.001). Mixed-effects analysis showed a significant difference in gene expression by drug dose (*p* < 0.001) and time point (*p = 0.001*) separately. In addition, there was an interaction effect between both factors (*p* = 0.011). Multiple comparison analysis revealed that in the high-dose MSG groups, treatment reduced ghrelin gene expression with a mean difference of – 1.722 for day 14 vs. day 7 (*q* value = 0.001) and a mean difference of – 2.325 for day 19 vs. day 7 (*q* value = 0.007) (Fig. 14).

## Discussion

### Morphological studies

In the present study, pregnant females in the different experimental treated groups showed a significant increase in body weight gain during the gestation period. This result agrees with Tala’a (2019), Mohamed et al. (2017), and Savcheniuk et al. (2014). Moreover, Abd El-Aziz et al. (2014) found prolonged administration of MSG causes an initial increase in weight gain followed by terminal suppression and independent of food consumption.

The results showed that MSG altered the distribution of implantation sites in the uterine horns in all treated groups. Abu Elnaga et al. (2019) found that MSG caused the unequal distribution of implantation sites in the uterine horns and the early death of embryos. George et al. (2013) reported that a low dose of MSG (0.4 g/kg b.w) reduced the number of rat embryos and some resorbed bodies appeared. Ibrahim et al. (2012) suggested that the early death of embryos may be due to an anemic condition induced by MSG as they found that hemoglobin level and red blood cell count were indicative of an anemic condition in pregnant rats treated with MSG. Moreover, the current study showed a significant reduction in maternal serum progesterone level at the 7th day of gestation which is considered a critical period after implantation time (Yoshinaga 2018) gives an idea about early preimplantation embryonic losses due to implantation failure where high progesterone level is needed.

The influence of various chemical agents on embryos and fetuses differs according to the ontogenetic stages and gestation period (Rugh 1968, Persaud 1979). The results showed that fetuses maternally treated with MSG exhibited some developmental malformations such as thin skin, a kinky tail, and abnormal hind limb. This was in agreement with Abu Elnaga et al. (2019) who observed the absence of eyelids, ear pinna, elongated and narrowed head, edema under the skin layer, atrophied eye, and abnormal fore and hind limbs in fetuses maternally treated with MSG (7 g/10 mL/kg b. wt) in the 9th to 14th days of gestation. Other different developmental malformations were observed in chick embryos (Roongruangchai et al. 2018; Al-Ghamdi 2017; Al-Qudsi and Al- Jahdali 2012) and zebrafish (Sajjaviriya et al. 2019).

Moreover, the recorded reduction in body weight and crown-rump length of fetuses at the 19th day of gestation maternally treated with MSG were in agreement with George et al. (2013), Tawfeeq and Jarjees (2013), Abu Elnaga et al. (2019), and Gad EL-Hak et al. (2021) in rat fetuses. This may be explained by Husarova and Ostatnikova (2013) who reported that MSG decreased the pituitary gland growth hormone level which is directly responsible for fetal growth. Also, Zanfirescu et al. (2019) suggested that the reduced fetus body weight may be due to MSG induce depression in the embryonic cells’ cellular differentiation process. Also, Al-Ghamdi (2017) suggested that MSG may directly affect embryo cell metabolism, as MSG can pass through the membrane easily and affect embryo development and growth. The present study revealed that the placental weight of MSG-treated pregnant rats was decreased compared with controls. This result was inconsistent with Abu Elnaga et al. (2019) who recorded that MSG-induced placental weight decrease in treated mothers. The later results were in harmony with the reduced placental ghrelin expression in the herein study, whereas placental ghrelin plays a pivotal role in the differentiation and growth of placental cells, modulates growth hormone production, and influences fetal development (Nakahara et al. 2006).

### Skeletal malformation

Many studies investigate MSG effects, but those dealing with MSG effects on the fetal skeleton are extremely limited. In the present study, the administration of MSG to pregnant rats caused a dose-dependent delay in chondrification and ossification of some parts of the axial and appendicular fetal skeleton. Dhindsa et al. (1978) found that MSG induced marked repression in the ossification of the developing endochondral bone and massive accumulation of adipose tissue with receded hemopoietic tissue within the bone marrow. They suggested that this may be due to MSG’s influence on the hydrolysis of alkaline phosphatase enzyme and glycolysis involved in the bone deposition or on secretions of hormones responsible for bone resorption.

### Histological studies

The current study showed that the liver of mothers treated with different doses of MSG was markedly affected. The fatty degeneration and lymphocytic inflammatory infiltration observed in the liver of MSG-treated mothers were in agreement with Kumbhare et al. (2015), Eid et al. (2018), Othman and Jumah (2019), and Gad EL-Hak et al. (2021). Kumbhare et al. (2015) stated that the presence of these fat droplets represents the response of hepatocytes to the ingestion of MSG, while Sherlock and Dooley (1993) suggested that the fatty changes induced in the liver in pathological conditions may be due to an imbalance between the normal rates of lipids synthesis and utilization. Shakoor et al. (1982) considered lymphocytic inflammation as a prominent response of body tissues facing any injurious impacts. According to Kumbhare et al. (2015), the migration of leukocytes to the site of inflammation is known as chemotaxis and can be explained the important role of lymphocytes as a prominent response of the body toward any injuries.

The observed hepatic necrosis (karyolysis, karyorrhexis, and pyknosis) in this study was in agreement with Aji et al. (2019), Shukry et al. (2020), El-Alfy et al. (2020), and Hamza and Diab (2020). Doaa et al. (2019) suggested that liver damage induced by MSG may be due to MSG accumulation inside the hepatocytes’ mitochondria; thus, the hepatocytes will not be able to maintain themselves metabolically or structurally, and this failure results in the death and dissolution of the cell.

Dilatation and congestion of the hepatic sinusoids, and central and portal veins recorded in this study were similar to results obtained by Hussin and Tala’a (2019) and Gad EL-Hak et al. (2021). According to Haschek et al. (2013), congestion may be due to the failure of the heart, which produces changes in different organs via excessive blood in the venous system and increases blood pressure on the surrounding capillaries, which may exert undue stress in the surrounding structures.

Among the important findings of the present study was the noticeable hyperplasia of bile ducts in high-dose MSG-treated mothers. This result agreed with Eid et al. (2018) and Gad EL-Hak et al. (2021). Hyperplasia associated with acute liver injury in which an increased number of ducts are confined to the portal tract is observed (Uchida et al. 1983; Nakanuma and Ohta 1986; Popper et al. 1987; Desmet 1987). According to Slott et al. (1990), hyperplasia of bile ducts indicated bile duct obstruction observed in forms of liver disease caused by infectious or toxic agents (Popper et al. 1987).

The liver of maternally treated fetuses showed many degenerative changes. This result is consistent with Al-Ghamdi (2017), Eid et al. (2018), and Gad EL-Hak et al. (2021). Eid et al. (2018) suggested that the toxic effect of MSG on the liver of rat pups is due to MSG, which causes highly distorted central veins and distorted blood vessels as well as reduce DNA total amounts in their liver. However, Al-Mosaibih (2013) considered that the toxic effect of MSG in the fetus’ liver is due to its oxidative damage in the liver.

MSG induced prominent lesions in the kidney tissues, and these observations were in agreement with Kumbhare et al. (2015), Mustafa et al. (2015), Shredah (2017), and Eid et al. (2019). Moreover, many studies showed a strong association between MSG exposure and renal effects (Eid et al. 2019, Yousef et al. 2019). Sharma et al. (2013) suggested that exposure to MSG may harm renal function which might be due to oxidative stress induced by MSG on the renal tissue. This can be explained by a change in the threshold of tubular reabsorption and glomerular filtration rate (El-Sheikh and Khalil 2011). Moreover, cells lining proximal convoluted tubules appeared to be the tissues in the kidney most highly sensitive to MSG. They suggested that this may be because they are the first to come in contact with the toxic agent after it is filtered by the glomeruli (Kumbhare et al. 2015, Dixit et al. 2014, Ortiz et al. 2006).

Fetuses’ renal atrophy and degeneration of Malpighian corpuscles were also noted by Andrew (2007), Tawfik and Al-Badr (2012), Osman et al. (2012), Elshama et al. (2016), and AL-Khatawi et al. (2019). Park et al. (2000) and Beyreuther et al. (2007) suggested that prenatal exposure to MSG would result in histological changes in the kidney because MSG readily crossed the placenta and accumulated in fetal tissue throughout gestation. On the other hand, Amin et al. (2010) and Inetianbor et al. (2015) suggested that MSG is capable of inducing oxidative damage to the brain, heart, liver, kidneys, and reproductive organs of developing fetuses by its effect on membranes, DNA, and antioxidant defense systems of cells.

### Biochemical parameters

The observed significant increases in serum ALT and creatinine in MSG-treated pregnant rats coincided with Tawfik and Al-Badr (2012), Osman et al. (2012), Sharma et al. (2013), and Gad EL-Hak et al. (2021). ALT is considered one of the cytosolic enzymes that are present exclusively in hepatocytes (Pratt and Kaplan 2000); thus, its presence in the plasma here denotes that there were deleterious effects on hepatocytes, as confirmed by histopathology that led to their damage and liberation of a such enzyme to plasma. Moreover, the elevated maternal ALP in MSG-treated pregnant mothers could be attributed to the hepatocytes function where the ALP is produced in case of biliary pressure. Moreover, it is closely related to the disturbances in hepatic secretory activities (Giannini et al. 2005, Kumar and Magon 2012). This result was consistent with the liver histopathology examination, which showed bile duct hyperplasia due to bile obstruction.

Progesterone is essential for the regulation of normal female reproductive functions. Also in the brain, its neurobehavioral expression is associated with sexual responsiveness (Genazzani et al. 2000) and in the bone prevention of bone loss (Balasch 2003). The present study showed that MSG decreased serum progesterone levels in MSG-treated pregnant rats at the 7th and 14th days of gestation. Similar results were obtained by Abu Elnaga et al. (2019) who reported that MSG increased the incidence of abortion after the reduction of progesterone hormone due to atrophic action of MSG on the ovary. In contrast at the 19th day of gestation, serum progesterone level showed a significant increase in the high MSG-treated group only. The progesterone level should be down-regulated at the end of the gestation period due to sensitization with estradiol receptors that prepare the uterus for oxytocin action and delivery (Leavitt et al. 1985). The herein results demonstrated that a high MSG-dose could impair the delivery and action of oxytocin due to failure of progesterone down-regulation at the end of gestation. This point may provide a future prospective research point.

The current study showed that treated groups showed down-regulation of Ghrelin in the placenta. Ghrelin is a gut hormone that activates its receptor, the growth hormone secretagogue receptor (GHS-R). Ghrelin controls bone formation and metabolism by modulating osteoblasts proliferation and differentiation (Delhanty et al. 2013, Pradhan et al. 2013). The observed delay in chondrification and ossification of some parts of the axial and appendicular fetal skeleton may be due to MSG-caused down-regulation of ghrelin in the placenta resulting in ghrelin disability to control fetal bone formation.

In conclusion, the present study demonstrated that pregnant rats treated with different doses of MSG at the 1st–18th days of gestation showed that MSG significantly induces many alterations in the morphological, histological, and physiological parameters under investigation. MSG was able to pass through the placenta and cause teratogenic effects on the developing fetuses as well as its pregnancy-threatening effect via altering progesterone levels. This study recommends that the uptake of MSG during pregnancy should be decreased to a minimal level due to its hazardous effects on pregnant mothers, which puts their fetuses at high risk of malformation.

## Data Availability

Data supporting findings are presented within the manuscript.
